# The Association of Il28b Genotype with the Histological Features of Chronic Hepatitis C Is HCV Genotype Dependent

**DOI:** 10.3390/ijms15057213

**Published:** 2014-04-25

**Authors:** Roberta D’Ambrosio, Alessio Aghemo, Raffaele De Francesco, Maria Grazia Rumi, Enrico Galmozzi, Stella De Nicola, Cristina Cheroni, Paul J. Clark, Guido Ronchi, Pietro Lampertico, Massimo Colombo

**Affiliations:** 1 Division of Gastroenterology and Hepatology, Ospedale Maggiore Policlinico, Università degli Studi di Milano, Milan 20100, Italy; E-Mails: alessio.aghemo@policlinico.mi.it (A.A.); enrico.galmozzi@gmail.com (E.G.); stella.denicola@gmail.com (S.N.); guido.ronchi@libero.it (G.R.); pietro.lampertico@unimi.it (P.L.); massimo.colombo@unimi.it (M.C.); 2 INGM, Istituto Nazionale Genetica Molecolare Milano, Milan 20100, Italy; E-Mails: defrancesco@ingm.org (R.F.); cheroni@ingm.org (C.C.); 3 Division of Hepatology, Ospedale San Giuseppe IRCCS Multimedica, Università degli Studi di Milano, Milan 20100, Italy; E-Mail: mariagrazia.rumi@unimi.it; 4 Duke Clinical Research Institute, Duke University, Durham, NC 27715, USA; E-Mail: drpjclark@gmail.com; 5 Kirby Institute for Infection and Immunity in Society, University of South Wales, Kensington 2033, Australia

**Keywords:** interleukin 28B (IL28B) polymorphism, hepatitis C virus (HCV), histology, liver biopsy, fibrosis, necroinflammation, steatosis, natural history

## Abstract

The interleukin 28B (IL28B) rs12979860 polymorphism is associated with treatment outcome in hepatitis C virus (HCV) genotype 1 and 4 patients. Its association with the histological features of chronic hepatitis C and disease severity needs further clarifications. To assess the correlation between IL28B genotype, HCV genotype and liver biopsy findings in untreated patients.

**Materials and Methods:**

Pre-treatment liver biopsies from 335 HCV Caucasian patients (59% males, age 50 years) enrolled in the MIST study were staged for fibrosis and inflammation according to the METAVIR and the Ishak scoring systems; steatosis was dichotomized as <5% or ≥5%. IL28B was typed by Taqman Single Nucleotide Polymorphism (SNP) genotyping assay. HCV genotype was 1 in 151 (45%), 2 in 99 (30%), 3 in 50 (15%) and 4 in 35 (10%) patients. IL28B genotype was CC in 117 (34%), CT in 166 (49%) and TT in 52 (15%). At univariate analysis, the IL28B CC genotype was associated with severe portal inflammation in HCV-1 patients (CC *vs.* CT/TT: 86% *vs.* 63%, *p* = 0.005), severe lobular inflammation in HCV-2 patients (CC *vs.* CT/TT: 44% *vs.* 23%, *p* = 0.03), and less fatty infiltration in HCV-1 patients (CC *vs.* CT/TT: 72% *vs.* 51%, *p* = 0.02). Despite the lack of any association between IL28B and fibrosis stage, in HCV-3 patients IL28B CC correlated with METAVIR F3–F4 (CC *vs.* CT/TT: 74% *vs.* 26%, *p* = 0.05). At multivariate analysis, the genotype CC remained associated with severe portal inflammation in HCV-1, only (Odds Ratio (OR): 95% Confidence Interval (CI): 3.24 (1.23–8.51)). IL28B genotype is associated with the histological features of chronic hepatitis C in a HCV genotype dependent manner, with CC genotype being independently associated with severe portal inflammation.

## 1. Introduction

The rs12979860 interleukin 28B (IL28B) gene single-nucleotide polymorphisms (SNPs) is one of the few genetic predictors proven to be of clinical utility since it identifies patients with hepatitis C infection caused by genotype 1 or 4 who undergo spontaneous [1,2] or treatment-induced [3–5] viral clearance [6–9]. In chronically infected patients, however, there is enough data to support the interplay between host and environmental factors in the achievement of a permanent response to interferon (*i.e.*, sustained virological response, SVR) including the stage of liver fibrosis, the degree of steatosis and of necroinflammation activity evaluated by liver biopsy. Interestingly, there is preliminary evidence for a heterogeneous distribution of histological markers of liver damage in patients with different IL28B genotypes suggesting an association between genetic control of interferon response and the natural course of chronic hepatitis C, thus extending our understanding of the IL28B functions beyond that of prediction of spontaneous resolution of acute hepatitis C virus HCV infection.

Scrutinizing a large set of liver biopsies taken in patients with chronic hepatitis C of any genotype who subsequently entered a pragmatic trial of pegylated interferon (PegIFN) and ribavirin (RBV) therapy, we aimed to explore any potential association between IL28B rs12979860 genotypes and the histological features of the liver in our patient population.

## 2. Results

**Figure 1.**
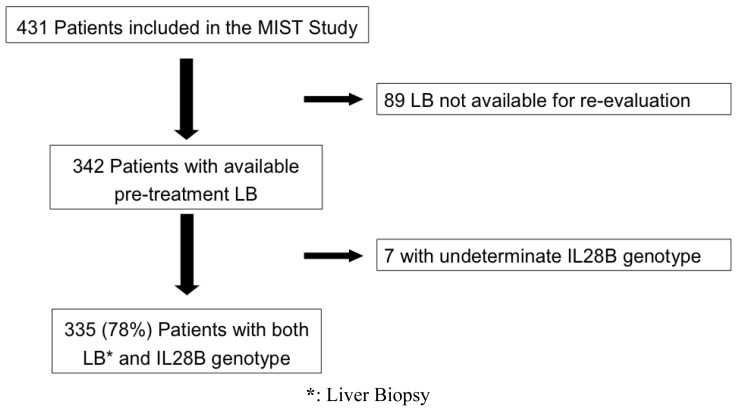
Patient enrollment according to the original MIST study.

Among the 431 patients originally included in the MIST study, 89 (21%) did not have a pre-treatment biopsy specimen available for re-evaluation; 7 (2%) had an undetermined IL28B genotype; 335 (78%) patients were ultimately analyzed (Figure 1). There were 197 (59%) males, the median age was 54 (21–73) years; liver disease was advanced (≥ F3) in 138 (41%). The HCV genotype distribution was 1 in 151 (45%), 2 in 99 (30%), 3 in 50 (15%) and 4 in 35 (10%). HCV-1 subtypes were not equally represented, since HCV-1b was present in 132 (87%) patients; therefore data from HCV-1a and HCV-1b were cumulatively analyzed. IL28B genotype was CC in 117 (35%) and CT/TT in 218 (65%) (Table 1). The median length of liver cores was 21 (12–54) mm; all LB were considered adequate for histological evaluation.

### 2.1. Fibrosis

**Figure 2.**
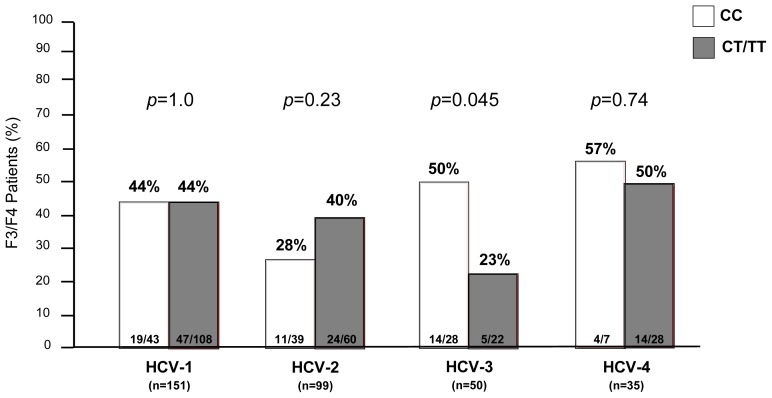
Prevalence of advanced fibrosis (F3–F4) according to IL28B genotype (CC *vs.* CT/TT).

According to METAVIR classification, fibrosis stage (F) was 0 in 10 (3%), 1 in 68 (20%), 2 in 119 (36%), 3 in 80 (24%) and 4 in 58 (17%) patients. F3–F4 fibrosis was uniformly distributed among the cohort with respect to IL28B genotype (CC *vs.* CT/TT: 41% *vs.* 41%). Following stratification by HCV genotype, F3–F4 was more prevalent in HCV-3 patients carrying the IL28B CC genotype than in HCV-3 carriers of the IL28B T allele (CC *vs.* CT/TT: 50% *vs.* 23%, *p* = 0.045). Other than HCV-3 genotype patients, no association between advanced liver disease (F3–F4) and IL28B genotype was found (CC *vs.* CT/TT: HCV-1 44% *vs.* 44%, *p* = 1.0; HCV-2 28% *vs.* 40%, *p* = 0.23; HCV-4 57% *vs.* 50%, *p* = 0.74) (Figure 2). Among patients with advanced fibrosis (F3–F4), those infected with HCV-3 had a higher prevalence of steatosis (74%) when compared to the remnants (HCV-1 43%, HCV-2 42%, HCV-4 49%; *p* < 0.0001). However, at univariate analysis steatosis did not emerge as predictor of advanced liver disease, since in HCV-3 patients AST and ALT values, IL28B CC genotype, fasting glucose levels and HOMA index were significantly associated with severe fibrosis. However, at multivariate analysis, AST values were independently associated with F3–F4 fibrosis, only (Table 2).

### 2.2. Inflammation

In the overall cohort we found no association between METAVIR histological activity of hepatitis and IL28B genotype distribution, and the same was true when patients were analyzed as single HCV-genotype category (Table 3), with the exception of more severe portal infiltrates in HCV-1 patients carrying the favorable IL28B CC genotype compared to other IL28B subgroups (CC *vs.* CT/TT: 86% *vs.* 63%, *p* = 0.005). In HCV-1 patients, univariate analysis identified older age and IL28B CC genotype as being associated to severe portal inflammation: both were confirmed by logistic regression analysis. Though HCV-2 patients with IL28B CC had more lobular inflammation (≥G3) than other IL28B subgroups (CC *vs*. CT/TT: 44% *vs.* 23%, *p* = 0.03), yet this association was not confirmed at multivariate analysis (Table 3). Finally, IL28B polymorphisms were not associated with intensity of piecemeal necrosis or presence of confluent hepatitis.

### 2.3. Steatosis

**Figure 3.**
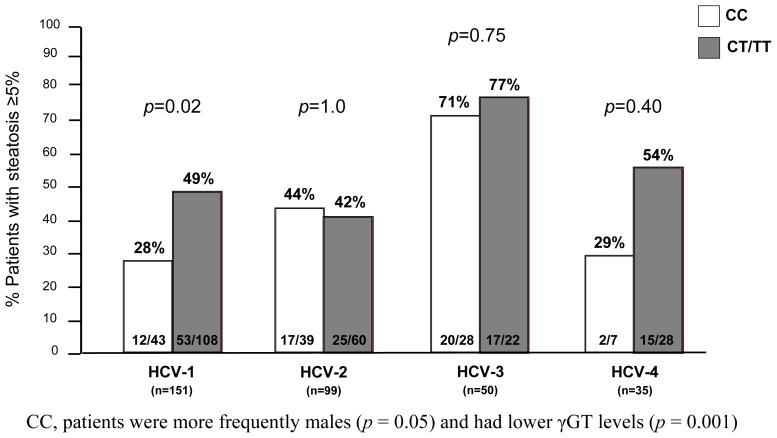
Prevalence of steatosis (≥5%) according to IL28B genotype (CC *vs.* CT/TT).

Steatosis was identified in 161 (48%) patients: 65 (40%) HCV-1, 42 (26%) HCV-2, 37 (23%) HCV-3 and 17 (11%) HCV-4. Overall, patients with steatosis had lower levels of cholesterol (166 *vs.* 175 mg/dL, *p* = 0.04) but higher levels of triglycerides (97 *vs.* 86 mg/dL; *p* = 0.004) than those without steatosis. In HCV-1 patients, the IL28B CC genotype was associated with less steatosis than other IL28B genotypes (CC *vs.* CT/TT: 28% *vs.* 49%, *p* = 0.02), whilst no association was found in patients carrying other viral genotypes (Figure 3). Among the several factors that at univariate analysis were associated with the presence of histological steatosis, γGT, Body Mass Index (BMI), HOMA index and triglycerides emerged as independent variables at logistic regression, only (Table 2). According to the lipid profiles, the IL28B genotype did not impact on either cholesterol (*p* = 0.08) or triglycerides values (0.13) in all viral genotypes; however, among patients with histological steatosis, patients carrying the CC allele showed slight lower values of triglycerides (CC *vs.* non-CC: 85 *vs.* 104, *p* = 0.049) but not of cholesterol (CC *vs.* non-CC: 173 *vs.* 165; *p* = 0.72).

## 3. Discussion

The scrutiny of a large cohort of unselected patients infected with HCV genotype 1 to 4 who had a pretreatment liver biopsy available to assess necro-inflammation activity, fibrosis and steatosis in the liver, did not reveal any association between IL28B genotype and these histological features of chronic hepatitis C. This notwithstanding, in HCV-1 patients portal inflammation was to some extent associated to host genetic background, as the IL28B genotype CC emerged as an independent predictor of severe portal inflammation together with advanced patient age.

This is an intriguing finding as HCV-1 patients are part of a cohort of early infected individuals in our community, represent the prevalent virus genotype population in Italy and are likely to better respond to IFN-based regimens that still represent the accessible form of anti-HCV therapy currently available in our country.

We are confident that our findings are unlikely to be biased by criteria of patient selection as the study was conducted in a large cohort of patients with chronic hepatitis C who were consecutively recruited and found eligible to PegIFN and RBV therapy according to internationally agreed criteria. Only a minority of patients (21%) who underwent a liver biopsy in other Centers (but were enrolled in the MIST study once we could confirm their eligibility after reviewing their biopsy slides brought to consultation) were excluded from this analysis. Importantly, the excluded population had a similar demography as the enrolled patients (data not shown). To further strengthen the robustness of our correlation study, we elected to assess necroinflammatory activity by both METAVIR and Ishak grading scores, with the hope that the latter would allow a more accurate definition of lobular topography of the necroinflammatory changes. Although we did not find any differences when stratifying the HAI scores obtained with the Ishak score by the IL28B genotype, the IL28B CC genotype was found to be associated to portal inflammation in HCV-1 as it was with lobular inflammation in HCV-2 patients. However, such an association was confirmed at multivariate analysis in HCV-1 patients only. Our findings, to some extent, corroborate a previous report of a correlation between histological features of liver inflammation and IL28B polymorphism in the overall HCV population [10]. In that study, in fact, though IL28BCC patients displayed higher total HAI scores (*p* = 0.007) as well as portal (*p* < 0.0001) and periportal (0.025) necroinflammation values than patients carrying the T allele, the lack of patient stratification by HCV genotype prevented demonstration of any virus specific correlation between histology and genes signaling interferon sensitivity. With all the caveats in the interpretation of portal infiltrate severity in our patients [11], portal inflammation could reflect the activation of the immune system against the infected hepatocytes which has been associated to the IFN sensitive IL28B genotype. IL28B CC patients, in fact, display a more pronounced activity of natural killer (NK) cells that are instrumental to viral clearance following treatments based on immunomodulatory drugs such as IFN and RBV [12,13]. Along this line are the findings of higher ALT values found in IL28BCC patients with intense histological necroinflammation due to HCV-1 [14], an observation built on the scrutiny of 1604 patients included in the IDEAL trial. Unfortunately, that study focusing on HCV-1 patients only missed any correlation analysis with the Ishak classification.

The correlation between Il28B and liver inflammation was the focus of another study in a cohort of untreated patients with HCV-2 and HCV-3 patients which revealed a significant association between IL28B CC genotype and portal inflammation in HCV-3 patients but not in HCV-2 patients [15]. In other words, these authors could not find in HCV-2 infected patients the same association we found between lobular necrosis and IL28B CC genotype.

We acknowledge, however, that the sample size of our HCV-3 group was small (15%), calling therefore for a cautious interpretation of our results, particularly considering that in the absence of any correlation between host genetic background and histological necroinflammation, the univariate analysis disclosed a statistically significant association between the IL28B CC genotype and liver fibrosis. Interestingly, these observations mimic the findings by Rembeck *et al.* of a trend for higher rates of liver fibrosis in IL28B CC patients with HCV-3 infection with respect to similar patients carrying the T allele [15]. In the thoroughly investigated Swiss cohort, Bochud and colleagues however failed to demonstrate any significant association between IL28B genotypes and fibrosis stage in patients HCV-3 using the rs860 SNP [16]. This could in part be explained by a direct cytoxic effect of HCV-3 strain on the liver as a consequence of its steatogenic and fibrosing activity: in HCV-3 infected patients, both a hepatic derangement of lipid metabolism and a faster progression of liver fibrosis have been demonstrated in comparison to other HCV genotypes [17,18]. In our cohort of patients, we found a higher prevalence of advanced fibrosis (F3–F4) in HCV-3 patients when compared to other genotypes. Whilst among patients with advanced fibrosis, those infected with genotype 3 displayed the highest prevalence of hepatic steatosis, in this subgroup of patients no differences in histological steatosis were found when comparing *F* < 3 and F3–F4 patients (71% *vs.* 74%; *p* = 0.74). Given that chronic infection with HCV in IL28B CC patients has been consistently associated with higher serum HCV-RNA values, one could speculate that viral replication is increased as a consequence of a host genetic background that might result into accelerated fibrosis of the liver in HCV-3 IL28B CC patients. Still, we acknowledge that liver fibrosis is a dynamic parameter that can hardly be assessed with accuracy by any cross-sectional investigation like ours.

More intriguing was the initial finding of a protective-like effect of IL28B CC genotype on the prevalence of histological steatosis in patients with chronic HCV-1 infection, which after adjustment for the other clinical features, was not confirmed by multivariate analysis. This is not a novel finding as steatosis was already found to prevail in carriers of the T allele in a cohort of 325 HCV-1 infected patients, an association that survived multivariate analysis [19]. In that study, IL28B CC patients had higher serum lipid levels that were thought to result from faster conversion of VLDL to LDL induced by the lipoprotein lipase activity. This enzyme in fact is suppressed by IFN-stimulating gene activity, which is down-regulated in patients carrying the CC genotype, causing low hepatic storage of lipids in the face of high serum levels of lipids [19]. As previously described, we also found higher levels of serum triglycerides in patients with steatosis, with patients carrying the T allele displaying the highest values. Although patients with steatosis showed slightly lower levels of total cholesterol, no differences were observed according to their IL28B genotype.

We acknowledge that our study has several limitations, one above all that the cross-sectional design that prevents the dynamic assessment of the histological end points while restricting the analysis to the subpopulation of patients selected for IFN therapy might limit transferability of our findings to the whole HCV population. We acknowledge also the unavoidable bias of correlations being obtained following the scrutiny of liver biopsy cores which is still the gold standard for evaluating liver fibrosis and inflammation in HCV, but at the same time may be challenged by high rates of inter-observer variability and sampling errors [20]. We do hope however that these latter drawbacks were attenuated by the fact that histological readings were carried out on liver cores of appropriate length by two independent experts who were blind to the demographic data.

In conclusion, this study provides the interesting finding that the segregation of IL28B genotypes is in accordance with different HCV related histological features in the liver.

## 4. Materials and Methods

### 4.1. Patient Population

This study was a sub-analysis of the MIST study, which was originally designed to compare the safety and effectiveness of PegIFNα2a and Peg-IFNα2b therapy with RBV in untreated patients with chronic hepatitis C (CHC) [21]. Patients who were enrolled in the original study had a pre-treatment liver biopsy (LB) and retrospectively consented to IL28B genotype testing (rs12979860). Exclusion criteria were co-infection with hepatitis B virus and/or human immunodeficiency virus, drug dependence, elevated alcohol intake, autoimmune hepatitis and/or any other liver disease and decompensated liver disease.

The protocol was approved by the Institutional Review Board of our Department and conformed to the ethical guidelines of the 1975 Declaration of Helsinki.

### 4.2. Laboratory Assessment

Baseline anthropometric measurements, including the height and weight for calculating the body mass index, were recorded. An overnight (12 h) fasting blood sample was taken for routine analysis (e.g., alanine aminotransferase (ALT), aspartate aminotransferase (AST), alkaline phosphatase (ALP), gamma-glutamyl transpeptidase (γGT), cholesterol, triglycerides, glucose and insulin. HOMA index was calculated according to the HOMA model formula, as previously described [22]. Serum HCV-RNA was quantified by Versant HCV-RNA 3.0 assay (bDNA 3.0; Bayer Corporation, Emeryville, CA, USA), with a sensitive limit of 615 IU/mL and a dynamic range from 615 to 7,700,000 IU/mL.

### 4.3. IL28B Genotyping

We analyzed the rs12979860 by real-time PCR on a LightCycler NANO instrument (Roche, Mannheim, Germany) using Taqman SNP genotyping assays according to manufacturers’ instructions (Applied Biosystems Inc., Foster City, CA, USA).

### 4.4. Histological Assessment

Liver biopsies (LBs) were performed with a 16-gauge Tru-cut needle (Uro-Cut 16G, TSK, Tokyo, Japan) and read by two independent pathologists (Roberta D’Ambrosio, Guido Ronchi) who were unaware of the patient’s IL28B genotype. Discrepancies were solved by consensus readings. LBs were considered adequate for histological analysis if they were longer than 10 mm and/or had at least 12 portal tracts. Liver fibrosis stage was evaluated semi-quantitatively according to the METAVIR scoring system [23,24]. Similarly, the severity of hepatic inflammation was evaluated according to both the METAVIR [24] and the Ishak [25] grading systems. Liver steatosis was recorded and dichotomized as absent (<5%) or present (≥5%).

### 4.5. Statistical Analysis

Statistical analyses were conducted using the Mann-Whitney *U* test or the Student’s *t*-test for continuous variables and the χ^2^ or Fisher exact probability test for categorical data. A probability value of *p* < 0.05 was considered statistically significant. All variables with statistical significance at the univariate analysis were included in the final model and odds ratios (OR) and corresponding 95% confidence interval (95% CI) were computed. Calculations were done with Stata 10.0 statistical package.

## 5. Conclusions

IL28B genotype is associated with the histological features of chronic hepatitis C in a HCV genotype dependent manner, with CC genotype being independently associated with severe portal inflammation.

## Figures and Tables

**Table 1.** Epidemiological and clinical characteristics of the cohort according to IL28B genotype.

Characteristics	Overall (*N* = 335)	IL28B CC (*N* = 117)	IL28B CT/TT (*N* = 218)	*p*-value
Males ∘	197 (59)	74 (63)	123 (56)	0.25
Age, years *	54 (21–73)	55 (26–72)	24 (21–73)	0.42
Body Mass Index *	25 (16–36)	25 (16–35)	25 (18–36)	0.91
HCV genotype ∘				
1	151 (45)	43 (37)	108 (50)	0.0008
2	99 (30)	39 (33)	60 (27)	
3	50 (15)	28 (24)	22 (10)	
4	35 (10)	7 (6)	28 (13)	
HCV-RNA, IU/mL **	774, 308	914, 086	743, 038	0.69
ALT, U/L *	101 (9–691)	113 (22–691)	96 (9–486)	0.042
γGT, U/L *	59 (11–633)	46 (12–633)	67 (11–502)	< 0.0001
Cholesterol, mg/dL *	172 (60–320)	174 (81–265)	170 (60–320)	0.08
Tryglicerides, mg/dL *	92 (40–380)	86 (40–340)	95 (65–380)	0.13
F3/F4 ∘	138 (41)	48 (41)	90 (41)	1.0
A2/A3 ∘	167 (50)	59 (50)	108 (46)	0.9

∘ N (%);

* Median (range);

** Median.

**Table 2.** Factors associated with the most relevant histological features according to viral genotypes at univariate and multivariate analysis. OR: Odds Ratio; CI: Confidence Interval.

HCV genotype	Histological features	Univariate analysis	Multivariate analysis	OR (95% CI)
HCV–1	Portal inflammation	Age	Age	1.04 (1.01–1.07)
		IL28B CC	IL28B CC	3.24 (1.23–8.51)

	Steatosis	Age	γGT	1.02 (1.01–1.03)
		Body weight	Body Mass Index	1.17 (1.03–1.33)
		Body Mass Index	HOMA Index	2.10 (1.03–4.27)
		AST	Tryglicerides	1.01 (1.00–1.02)
		γGT	-	-
		IL28B CT/TT	-	-
		Diabetes	-	-
		Glucose	-	-
		HOMA Index	-	-
		Tryglicerides	-	-

HCV–2	Lobular inflammation	AST	AST	1.01 (1.00–1.01)
		ALT	-	-
		γGT	-	-
		IL28B CC	-	-

HCV–3	Fibrosis	AST	AST	1.07 (1.02–1.11)
		ALT	-	-
		IL28B CC	-	-
		Glucose	-	-
		HOMA Index	-	-

**Table 3.** Necroinflammation according to HCV genotype and IL28B (CC *vs.* CT/TT).

Grading	HCV-1		HCV-2		HCV-3		HCV-4	

	CC (*n* = 43)	CT/TT (*n* = 108)	CC (*n* = 39)	CT/TT (*n* = 60)	CC (*n* = 28)	CT/TT (*n* = 22)	CC (*n* = 7)	CT/TT (*n* = 28)
METAVIR A ≥ 2	49%	50%	54%	48%	43%	45%	71%	54%
Ishak								
Piecemeal ≥ 3	23%	13%	28%	18%	14%	10%	14%	18%
Portal ≥ 3	86%	63%	69%	65%	50%	55%	71%	46%
Lobular ≥ 3	19%	19%	44%	23%	21%	36%	71%	29%
Confluent ≥ 3	23%	5%	18%	7%	14%	5%	0	14%

*p*-values ns for all comparisons except for severe portal inflammation in HCV-1 (*p* = 0.005) and severe lobular inflammation in HCV-2 (*p* = 0.03).
